# Timed intercourse in association with controlled ovarian hyperstimulation as the first-line treatment of couples with unexplained subfertility

**DOI:** 10.5935/1518-0557.20220001

**Published:** 2022

**Authors:** Moacir Rafael Martins Radaelli, Vânia Cibelle Mingetti-Câmara, Raul Nalano, Nathan Ichikawa Ceschin, Paula Motta Almodin Cerialle, Carlos Gilberto Almodin

**Affiliations:** 1 Materbaby Reprodução Humana e Genética, Maringá, Brazil; 2 Departamento de Urologia, Faculdade de Medicina Ingá, UNINGÁ, Maringá, Brazil; 3 Clínica de Reprodução Humana FERTICLIN, São Paulo, Brazil; 4 Felicitta Instituto de Fertilidade, Curitiba, Brazil

**Keywords:** assisted reproductive technologies, timed intercourse, controlled ovarian hyperstimulation, swim-up

## Abstract

**Objective:**

To report on the pregnancy outcomes of timed intercourse (TI) with controlled ovarian hyperstimulation (COH) as the first-line treatment of unexplained subfertility, and provide some evidence on the factors involved.

**Methods:**

The records of couples treated between January 2016 and March 2019 were retrospectively analyzed. Couples were selected for TI based on standard infertility evaluation. Semen analysis by swim-up was conducted and the total motile sperm count (TMSC) obtained. The main outcome measured was the clinical pregnancy rates. Data were analyzed with t test, Pearson’s Chi-squared test, and the Wald test for logistic regression with *p*≤0.05.

**Results:**

The records of 275 couples (449 cycles) were included in the analysis. Patients underwent TI up to six attempts. Patient- and cycle-based pregnancy rates were 18.55% and 13.14%, respectively. Eight patients got pregnant twice, resulting in a cumulative pregnancy rate of 21.4%. Women that did not get pregnant demonstrated a statistically higher mean age value than women who did (p=0.0186). Logistic regression indicated that for every year added to the woman’s age, the chances of pregnancy reduced by 6.45%, and for cycles with TMSC ≥ 5 million, the chances of pregnancy were 1.91 times higher when compared to TMSC < 5 million.

**Conclusions:**

TI with COH should be considered as the first-line treatment for selected couples with unexplained subfertility before more traumatic and costly IVF treatments were considered. The findings can assist doctors to conduct a more educated counselling concerning the chances patients have to get pregnant with TI.

## INTRODUCTION

Subfertility, usually defined by the inability to conceive within 12 months of routine unprotected sexual intercourse, is a common condition that affects around 10% to 15% of couples (Practice Committee of the American Society for Reproductive Medicine, 2020). Among these, approximately 15% of couples present no apparent reason for not conceiving and are considered to have unexplained subfertility (Quaas & Dokras, 2008; Practice Committee of the American Society for Reproductive Medicine, 2020). Among the factors that may prevent couples to conceive is the incorrect timing of intercourse. Because both the sperm and the oocyte have a limited life span, identifying the ovulatory period can increase the chances of couples with subfertility to conceive and reduce costs with unnecessary procedures (Manders *et al*., 2015). One or two days before ovulation has been suggested as the best timing for intercourse (Stanford *et al*., 2002).

Among all the assisted reproductive technology (ART) procedures available to couples wishing to have children, timed intercourse (TI) during the fertile period is the simplest, the least traumatic, and the most inexpensive approach (Agarwal & Haney, 1994). Detection of urinary luteinizing hormone (LH) surge, estrogen measurements, body temperature follow-up, cervical mucus investigation, calendar monitoring, and ultrasonography can all be used to identify ovulation and define TI (Manders *et al*., 2015). TI may also be conducted in association with controlled ovarian hyperstimulation (COH), which permits not only to increase the number of mature follicles and trigger ovulation to achieve the best timing for TI, but also to improve the quality of the endometrium (Guzick *et al*., 1998; van Rumste *et al*., 2008).

Apart from ovulation management, the quality and quantity of sperm is another major factor to affect outcomes (Horvath *et al*., 1989). Testing the quality of the sperm can assist counselling couples on their chances of success before a mutual decision can be made on the initial treatment procedure (van Weert *et al*., 2004). Controversy, however, still exists in regard to the normal seminal parameters defined by the WHO, which have been considered insufficient to define male fertility potential and, as a result, the treatment strategy to be offered to couples (Haugen *et al*., 2006). Apart from the obvious psychological, economic, and medical implications, geographical factors may also play a role in subfertility (Evers, 2002; Gnoth *et al*., 2005). Thus, the average parameters and results concerning male subfertility should be carefully investigated for each population (Fisch & Goluboff, 1996).

Large reproductive centers often demonstrate little interest in low complexity techniques such as TI and intra-uterine insemination (IUI). Despite the recognizable benefits to patients in terms of the risks and financial costs over *in vitro* fertilization (IVF), many clinics consider IVF and intracytoplasmic sperm injection (ICSI) as the first therapeutic options for couples wishing to get pregnant (Miskry & Chapman, 2002; Bhatt & Baibergenova, 2008; Manders *et al*., 2015). However, apart from being significantly more expensive and invasive, there is no guarantee of success with the use of these techniques (European IVF-Monitoring Consortium & European Society of Human Reproduction and Embryology, 2016). TI is a more patient-friendly approach that involves minimum intervention and offer the opportunity for couples with unexplained subfertility and mild male factor to achieve pregnancy before considering moving to more complex and expensive IVF techniques. Despite some adverse aspects associated with TI, such as the delay and stress caused by the interference with natural coital habits (NICE, 2013), these should not be seen as an impediment, or an excuse, for not attempting the procedure. Before a decision can be made, however, options should be thoroughly discussed with the couple based on a thorough clinical assessment, educated information on the different factors that may influence the success rates, costs, as well as possible risks and benefits involved in the course of the infertility treatment (Pennings & Ombelet, 2007).

Therefore, the objective of this study was to report on the pregnancy outcomes obtained with the use of TI with COH as the first line of treatment of a large group of Brazilian couples diagnosed with unexplained subfertility and provide some evidence on the factors involved.

## MATERIALS AND METHODS

This retrospective observational study was conducted based on the analysis of the records of couples who had been unsuccessfully trying to conceive naturally for at least 1 year and, after counselling, decided to undergo timed intercourse (TI) with COH at a private fertility clinic in the city of Maringá, Brazil, between January 2016 to March 2019.

The study was conducted in conformance with the ethical standards of to the Resolution 466/2012 of the Brazilian National Health Council, the 1964 Helsinki declaration and its later amendments, and the recommendations set by the Strengthening the Reporting of Observational Studies in Epidemiology (STROBE) guidelines (von Elm *et al*., 2014).

The study was approved by the local Institutional Review Board. Because of the retrospective nature of the study, no informed consent was required.

### Sample

All couples underwent a standard infertility evaluation, which included medical history, physical examination, tubal patency by hysterosalpingography, confirmation of an ovulatory cycle by midluteal serum progesterone, and routine semen analysis. All male partners underwent at least one semen collection by masturbation for the swim-up test and analysis.

### Inclusion criteria

The inclusion criteria for female partners were: i) good ovarian reserve as assessed by the anti-Mullerian hormone (AMH) > 2 ng/ml, follicle stimulating hormone (FSH) < 10 ng/ml, and antral follicles count > 8; ii) no obstructive female factors; iii) no serious female pathologies; iv) no history of genetic diseases in the family; and v) no apparent indication for IVF.

The inclusion criteria for male partners were: i) comfort with their sexual performance; and ii) being capable of collecting semen for analysis through masturbation. Men that had previously undergone clinical or surgical treatments to correct abnormalities were also included. Male factor etiology was not an exclusion factor, but men presenting genetic factors that required preimplantation genetic testing for aneuploidies (PGTA) were excluded.

### Swim-up

The collected semen was liquefied at room temperature for 30 to 45 minutes. In case liquefaction did not occur, the semen was passed through a syringe and needle. A 1.0 ml volume of liquified semen was gently deposited at the bottom of a round-bottomed test tube under 2 ml of buffered medium (HEPES, Ingamed, Brazil) which had been previously warmed in an incubator at 37^o^C and 6% CO_2_ for 30 min. The spermatozoa were allowed to swim up from the semen to the surface of the medium for 60 minutes inside the incubator. At the end of the incubation period, 1.3 ml of the supernatant buffered medium was gently aspirated, transferred to a centrifuge tube, and centrifuged at 1500 rpm for 5 min. The resulting pellet was resuspended in 0.5 ml of HEPES medium and homogenized for analysis.

Sperm concentration and motility were determined with a Makler counting chamber both before and after the swim-up. Motile sperm were classified in GI (the spermatozoa moved without leaving its place), GII (the spermatozoa presented progressive, but non-linear movement), and GIII (the spermatozoa presented progressive linear movement). GIII motile sperm after swim-up were counted in 3 rows with 10 quadrants each, and the average result expressed in term of the total motile sperm count (TMSC) in millions per ml.

### Ovulation induction and TI

Transvaginal ultrasonography (TV-US) was performed between the first and, at most, the third day of the menstrual cycle and, if no residual follicle <12 mm was observed, medication was initiated. Patients received clomiphene citrate (Clomid^®^ - Sanofi Aventis, Brazil) 50 mg twice daily from the third to the seventh day of the cycle, associated with estradiol valerate (Prymogina^®^, Bayer, Brazil) 2 mg twice daily. Based on patient’s ovarian reserve, gonadotropin 75 IU (Gonal^®^, Merck, Brazil), was administered subcutaneously 2 to 8 units per cycle during induction. On the eighth day of the cycle, the male partner was asked to ejaculate through masturbation to renew his sperm stock. From the 10^th^ day of the menstrual cycle, monitoring was started to define the patient’s ovulation and synchronize with the sperm reserve peak (3 to 5 days of abstinence). On the 10^th^ day of the cycle, TV-US was performed to assess endometrial development, and to synchronize ovulation and endometrium development (>7 mm) the day before the coitus. If the follicles had not reached the size of 18 to 20 mm, new doses of gonadotropin 75 IU were administered and, if required, a new TV-US assessment was scheduled. In the same period, if the endometrium had not reached the minimum thickness of 7 mm, estradiol valerate was increased to 6 mg to 8 mg/day. As soon as the follicles reached 18 to 20 mm in diameter, hCG (Choriomon^®^, UCB, Brazil) 5,000 IU was administered subcutaneously to trigger ovulation, and TI was scheduled for 36 to 40 hours later. In case four primary or more follicles were observed after induction, the TI cycle was simply cancelled or the oocytes collected and vitrified. A post-coital TV-US was conducted 10 to12 hours after TI to observe follicular rupture. From this moment, patients continued to use estradiol valerate at the induction dose and started vaginal progesterone (Utrogestan^®^, Capsugel Ploermel, France) 200 mg twice a day. Fourteen days after TI, β-hCG was measured to verify pregnancy. No limits were established to the number of TI cycles couples could undergo.

### Outcome variable

The main outcome measured was the clinical pregnancy rates, assessed with β-hCG ≥ 25 mIU/ml 14 days after TI, which was confirmed by the presence of an intrauterine gestational sac by TV-US at 6 weeks of gestation.

### Statistical analysis

Descriptive statistics was used to show the distribution of cycles/patients, cancelled cycles/patients, cycle-based and patient-based pregnancy rates, as well as the means and standard deviation (SD) for the age of patients, FSA and AMH levels, and the TMSC concentrations.

Furthermore, the t test was used to compare the mean values for age, FSH, HAM and TMSC for all 449 observations (cycles) between pregnant and non-pregnant patients. Pearson’s Chi-squared test was used to verify possible associations between the distribution of cycles according to different age groups and TMSC groups and the occurrence of pregnancy. The Wald test for logistic regression was used to analyze the chances for the occurrence of pregnancy, taking into account the variables available in the database and the associations between potential explanatory variables and the occurrence of pregnancy. A binary response variable indicating the occurrence of pregnancy (value = 1 if the cycle resulted in pregnancy, and value = 0 if it did not) was used. The estimate of the logistic model considered all 449 cycles, and included cycles canceled along with cycles that did not result in pregnancy. The model also made use of the cluster option per patient, which indicates that the observations (cycles) that refer to the same patient are not independent of each other, i.e., it considers that the result of one cycle is not independent of the result of another cycle for the same patient.

The analyses were performed using the R package version 3.6.1 (R Core Team, 2019 - R Foundation for Statistical Computing, Vienna, Austria) with *p*≤0.05.

## RESULTS

A total of 275 patients who underwent a total of 449 cycles were involved in the study. Participating patients underwent up to six consecutive attempts at pregnancy. No patient presented multiple pregnancies at any of the cycles.

Of the 449 cycles, 52 (11.58%) were cancelled, of which 38 (73.1%) due to failed intercourse at the allocated time, and 14 (26.9%) to exceeding number of mature follicles (≥4) after COH. Pregnancies were registered in 59 cycles (13.14%). When the number of cancelled cycles was removed from the analysis, the overall cycle-based pregnancy rate increased to 14.86% (Table 1).

**Table 1:** Distribution of cycles, cancelled cycles, and cycle-based pregnancy rates at each consecutive attempt.

Attempts	Cycles		Cancelled cycles		Pregnancies		
	N.	%	N.	%	N.	%	%*
**1**	155	34.52%	19	12.26%	26	16.77%	19.12%
**2**	152	33.85%	18	11.84%	20	13.16%	14.93%
**3**	111	24.72%	7	6.31%	7	6.31%	6.73%
**4**	20	4.45%	3	15.00%	4	20.00%	23.53%
**5**	5	1.11%	3	60.00%	0	0.00%	0.00%
**6**	6	1.34%	2	33.33%	2	33.33%	50.00%
**Total**	449	100.00%	52	11.58%	59	13.14%	14.86%

* Excluding cancelled cycles.

Of the 275 women, 51 (18.55%) became pregnant at least once. Eight patients got pregnant twice; 7 women tried a new attempt after a miscarriage, and one woman tried a second baby. A total of 42 women (84%) got pregnant after undergoing one or two cycles. No pregnancy was observed in the woman that underwent five cycles. The cumulative pregnancy rate, taking into consideration women that got pregnant twice (59 pregnancies to 275 patients), was 20.99%. The number of canceled patients is proportional to the number of cycles performed by the patients. Thus, the 18 cycles canceled by patients who performed a total of 2 cycles are equivalent to 9 canceled patients (18 cycles canceled/2 cycles in total = 9 patients); similarly, the 2 cycles canceled by the patient who performed 6 cycles in total is equivalent to 0.33 cancelled patient (Table 2).

**Table 2:** Distribution of patients, cancelled patients, and patient-based pregnancy rates at each consecutive attempt.

Attempts	Patients		Cancelled patients			Pregnancies	
	N.	%	N.^1^	%	N.	%	%*
**1**	155	56.36%	19.00	12.26%	26	16.77%	19.12%
**2**	76	27.64%	9.00	11.84%	16	21.05%	23.88%
**3**	37	13.45%	2.33	6.31%	6	16.22%	17.31%
**4**	5	1.82%	0.75	15.00%	2	40.00%	47.06%
**5**	1	0.36%	0.60	60.00%	0	0.00%	0.00%
**6**	1	0.36%	0.33	33.33%	1	100.00%	150.00%
**Total**	275	100.00%	32.02	11.64%	51	18.55%	20.99%

1 Proportional number: cycles canceled/total cycles.

* Excluding cancelled cycles.

Overall, the mean age of the participating women in the 449 cycles was 33.24 years (±3.918). Mean age of all 275 women who completed the first cycle was 33.44 years (±4.037) (Table 3).

**Table 3:** Means and standard deviation (SD) values for the age of patients (years), FSA (ng/ml), AMH (ng/ml) and TMSC concentration (million/ml) obtained with the swim-up test for the male partners at each attempt.

Attempt	N. cycles	Age		FSH		AMH		TMSC	
		Mean	SD	Mean	SD	Mean	SD	Mean	SD
**1**	155	33.44	4.0371	6.83	2.5067	3.46	3.0681	5,381,091	6,553,781
**2**	152	32.93	3.8917	6.32	1.9667	3.90	3.0486	5,588,333	6,314,119
**3**	111	33.32	3.3603	6.41	1.8664	3.84	3.1720	6,022,727	7,776,885
**4**	20	31.29	2.7516	5.31	0.9542	5.47	5.1194	3,071,429	1,509,651
**5**	5	30.00	1.4142	5.52	0.2404	9.15	10.2531	1,750,000	1,767,767
**6**	6	31.00	.	8.34	.	9.60	.	500,000	.
**Total**	449	33.24	3.9180	6.63	2.3046	3.68	3.1757	5,436,303	6,553,359

FSH=follicle stimulating hormone; AMH=anti-Mullerian hormone; TMSC=total motile sperm count.

Test t demonstrated a statistically higher mean age value for women who did not get pregnant when compared with those who did (*p*=0.0186). The mean AMH value for women who got pregnant was statistically higher when compared with women who did not (*p*=0.0112). However, no significant differences in the mean FSH and TMSC concentration values were observed between the two groups (Table 4).

**Table 4:** Comparison between the means and standard deviation (SD) values found for the age (years), FSH (ng/ml), HAM (ng/ml) and TMSC (million/ml) of patients who did not get pregnant and those who did.

Variable	Overall	Non-pregnant + cancelled	Pregnant	*t* teste	*p*-value
**Age**	33.23 (0.184)	33.40 (0.199)	32.12 (0.467)	2.3626	0.0186*
**FSH**	6.62 (0.108)	6.63 (0.120)	6.55 (0.239)	0.2605	0.7946
**HAM**	3.68 (0.149)	3.53 (0.155)	4.66 (0.476)	-2.5469	0.0112*
**TMSC**	5436303 (309272.1)	5354615 (338800.5)	5976271 (726489.4)	-0.6787	0.4977
**Total**	449	390	59	.	.

FSH=follicle stimulating hormone; AMH=anti-Mullerian hormone; TMSC=total motile sperm count.

* Statistically significant, *t* test.

Female patients were aged between 22 and 45 years old. The youngest woman to get pregnant was aged 23 years, while the oldest was 40 years. In most cycles, patients were aged between 30 and 39 years. Of the 390 cycles that did not result in pregnancy, 39% were conducted in women aged between 35 and 39 years. Of the 59 cycles that resulted in pregnancy, 50.8% was conducted in patients aged between 30 and 34 years. Pearson’s Chi-squared test indicated that there was a statistically significant association (*p*=0.038) between the different age groups and the occurrence of pregnancy. Of the 87 cycles in which patients were up to 29 years old, 16.1% resulted in pregnancy, while of the 172 cycles in which the patients were aged between 30 and 44 years, 17.4% resulted in pregnancy (Table 5).

**Table 5:** Distribution of cycles per age group and the occurrence of pregnancy.

Age group	Cycles		Non-pregnant + cancelled		Pregnant		Pregnancy rate per age group
	N.	%	N.	%	N.	%	
**Up to 29 years**	87	19.4%	73	18.7%	14	23.7%	16.1%
**30 to 34 years**	172	38.3%	142	36.4%	30	50.8%	17.4%
**35 to 39 years**	166	37.0%	152	39.0%	14	23.7%	8.4%
**40 years +**	24	5.3%	23	5.9%	1	1.7%	4.2%
**Total**	449	100.0%	390	100.0%	59	100.0%	13.1%

Pearson´s Chi^2^(3)=8.5271; *p*-value=0.038.

The swim-up test demonstrated TMSC concentrations ranging from 100,000/ml to 40,000,000/ml. Pregnancy was observed with a minimum TMSC = 400,000/ml and a maximum TMSC = 25,300,000/ml. In most cycles, male partners presented TMSC ≥ 5 and < 10 million/ml, while TMSC ≥ 20 million/ml was observed in just 22 cycles. Pearson’s Chi-squared test demonstrated no statistically significant associations (*p*=0.263) between the different TMSC groups and the occurrence of pregnancy. Of the 80 cycles in which male partners presented TMSC < 1 million/ml, 8.8% resulted in pregnancy, while of the 111 cycles with TMSC ≥ 5 and < 10 million/ml, 18.9% resulted in pregnancy (Table 6).

**Table 6:** Distribution of cycles per TMSC group (million/ml) and the occurrence of pregnancy.

TMSC	Cycles		Non-pregnant + cancelled		Pregnant		Pregnancy rate per TMSC group
	N.	%	N.	%	N.	%	
**< 1**	80	17.8%	73	18.7%	7	11.9%	8.8%
**≥ 1 and < 2**	69	15.4%	63	16.2%	6	10.2%	8.7%
**≥ 2 and < 3**	43	9.6%	36	9.2%	7	11.9%	16.3%
**≥ 3 and < 4**	49	10.9%	43	11.0%	6	10.2%	12.2%
**≥ 4 and < 5**	23	5.1%	22	5.6%	1	1.7%	4.3%
**≥ 5 and < 10**	111	24.7%	90	23.1%	21	35.6%	18.9%
**≥ 10 and < 20**	52	11.6%	43	11.0%	9	15.3%	17.3%
**≥ 20**	22	4.9%	20	5.1%	2	3.4%	9.1%
**Total**	449	100%	390	100.0%	59	100.0%	13.1%

TMSC=total motile sperm count.

Pearson’ Chi^2^ (7)=8.8635; *p*-value=0.263

However, when TMSC values were analyzed as binary variables, Pearson’s Chi-squared test indicated that TMSC ≥ 5 million/ml was significantly associated with the occurrence of pregnancy (0.029). While pregnancy occurred in 10.2% of the 262 cycles with TMSC < 5 million/ml, pregnancy occurred in 17.3% of the 185 cycles with TMSC ≥ 5 million/ml (Table 7).

**Table 7:** Binary distribution of cycles per TMSC group (million/ml) and the occurrence of pregnancy.

TMSC	Cycles		Non pregnant + cancelled		Pregnant		Pregnancy rate per TMSC group
	N.	%	N.	%	N.	%	
< 5	264	58.8%	237	60.8%	27	45.8%	10.2%
≥ 5	185	41.2%	153	39.2%	32	54.2%	17.3%
Total	449	100.0%	390	100.0%	59	100.0%	13.1%

TMSC=total motile sperm count.

Pearson’s Chi^2^ (1)=4.7637; *p*-value=0.029.

Because FSH did not show a statistically significant association with the probability of pregnancy, it was not included in the logistic regression model. Conversely, because the variables age and AMH showed statically significant association with the chance of pregnancy occurring during a cycle, they were included in the model as controls. The variable of interest TMSC concentration (million/ml) did not present a significant relationship with the chance of pregnancy in continuous form, neither when the variable was divided into 8 categories. Thus, the best solution was to divide the variable into two categories (< 5 and ≥ 5 million/ml). This binary variable was selected for the regression model.

According to the Wald test, the model as a whole was statistically significant (*p*=0.0036) with age at ≤ 10% significance, and AMH and TMSC at ≤ 5% significance. In general, controlling for the other variables, age was negatively associated with the probability of pregnancy. On the other hand, AMH levels and TMSC concentrations ≥ 5 million were positively associated with the chances of pregnancy. Thus, for every year added to the woman’s age, the chances of pregnancy were reduced by 6.45%. For every additional unit of AMH, the chances of pregnancy increased by 8.30%. For cycles with TMSC concentrations ≥ 5 million/ml, the chances of pregnancy were 1.91 times higher when compared to TMSC concentrations < 5 million/ml (Table 8).

**Table 8:** Odds ratio for the occurrence of pregnancy for the variables age (years). AMH (ng/ml), and TMSC (million/ml), according to logistic regression model.

Pregnancy	Odds Ratio	SE^1^ (robust)	z	P>z	95% Confidence Interval	
Age	0.9355	0.03293	-1.90	0.058	0.87311	1.00228
HAM	1.0830	0.03893	2.22	0.027	1.00931	1.16202
TMSC ≥ 5	1.9095	0.56902	2.17	0.030	1.06482	3.42433
Constant	0.7207	0.86876	-0.27	0.786	0.06788	7.65231

AMH=anti-Mullerian hormone; TMSC=total motile sperm count.

Wald test for logistic regression of 449 observation (cycles)

SE=Standard error adjusted for 275 clusters (patients)

Wald Chi^2^ (3)=13.52; *p*=0.0036

## DISCUSSION

This study was conducted in order to provide some data on different aspects involving in the use of TI with COH as a first-line treatment for couples with unexplained subfertility. The results demonstrated that 18.55% of the participating women were able to get pregnant, some of them twice. Although the data suggest that younger female patients and male partners with TMSC ≥ 5 million/ml seem to benefit most from the treatment, no definitive limits both in terms of age and TMSC, which would contraindicate the treatment, seems to exist. Likewise, although the results show that most pregnancies occurred after the first or the second attempt, pregnancies were also observed in patients that underwent three or more attempts.

The patient-based pregnancy rate and cycle-based pregnancy rate (18.55% and 13.14%, respectively) obtained in the present study are similar to those previously found (22.1% and 7.89%, respectively) for women with polycystic ovarian syndrome undergoing TI (Abu Hashim *et al*., 2011). Eight women in the sample got pregnant for a second time, seven of them after having their pregnancies interrupted, while one attempted a second baby. These represent a cumulative cycle-based pregnancy rate of 20.99%. Despite the significant results observed, pregnancy rates could have been even higher had it not been for the number of cancelled cycles, which represented 11.58% of the sample. In the present study, a total of 38 cycles (8.5%) were cancelled due to failed intercourse at the allocated time. Under the perspective of the obligation to have intercourse at an established date, the psychological welfare and performance of male partners can be seriously affected. In a previous study, erectile dysfunction affected 42.8% of men in a TI program (Bak *et al*., 2012). The relatively low number of failed intercourses seen in the present sample may probably be attributed to the support provided to couples during counselling. Nevertheless, this is an inherent problem to TI, which can be minimized but not completely avoided.

COH is an important tool at our disposal to increase the number of oocytes available during coitus and improve the chances of conception with TI. The drawback with this strategy, however, concerns the inherent risk of multiple pregnancies (van Rumste *et al*., 2008). These can be minimized without compromising pregnancy rates with the use of a mild COH program in association with careful cycle monitoring and strict cancellation criteria (te Velde & Cohlen, 1999). Thus, a very mild stimulation protocol with clomiphene citrate was used (Practice Committee of the American Society for Reproductive Medicine, 2013), and any cycle presenting more than 3 mature follicles after induction were also cancelled (van Rumste *et al*., 2008). As a result, no cases of multiple pregnancies were observed in any of the patients.

The number of attempts can represent an important strategy in TI to increase the chances of pregnancy. Although no strict limits are established in our clinic for how many attempts couples can undertake, it is not common for couples to undergo more than two or three, as they may feel that the procrastination and stress involved may be unbearable (te Velde & Cohlen, 1999). In fact, the results showed that most patients in the present sample (97.5%) conducted up to three attempts, when 89.8% of all pregnancies occurred. Had some of the couples insisted further, they could have increased their chances to get pregnant, as observed in the present study.

Female partner age is always an important indicator for the chances of pregnancy in general. As expected, most pregnancies (76.47%) occurred in younger women aged up to 34 years, indicating that this age group seem to benefit more from TI than older women. The logistic regression analysis clearly demonstrated that for every year added to the woman’s age, the chances of pregnancy were reduced by 6.45%. This is an important information on which counselling can be based.

Male fertility factor is also a significant issue for couples who are candidates for TI. The total motile sperm count (TMSC) has been considered one of the main prognostic factors to increase pregnancy rates when low complexity techniques such as TI and intrauterine insemination (IUI) are considered (Miller *et al*., 2002; Zhao *et al*., 2004). In a review of the literature, TMSC cut-off values of 5 million were established for the conduction of IUI in three papers and of 10 million in six papers (Ombelet *et al*., 2014). Seminal prewash parameters are not the same after seminal processing, and the sperm should be better evaluated by more specific techniques, such as swim-up (Ombelet *et al*., 2014). Swim-up is a sperm selection technique used to isolate sperm with the greatest potential for fertilization intended to increase gamete density at the site of fertilization during IUI (Cohlen, 2005). After swim-up, TMSC concentrations of at least 1 million/ml has been suggested as an important and substantial discriminative performance in IUI programs (Ombelet *et al*., 1997).

It would be fair to imagine that the same parameters usually indicated for IUI could also be applied to TI. As part of their diagnostic workup, all male partners undergo semen analysis with swim-up to assist counselling and the definition of the strategy to be adopted. However, provided that some motility is observed in the test, no lower limits are established for those who want to attempt TI. Patients with TMSC as little as 100,000/ml were present in the sample, and a pregnancy was observed with TMSC = 400,000/ml. This finding is in agreement with Ombelet *et al*. (2014), who suggested that the TMSC cut-off value of 1 million/ml is just an indication that above this level success rates seem to be higher, but it does not necessarily mean that pregnancy cannot be achieved with TMSC below this level. As a matter of fact, no statistically significant associations with the occurrence of pregnancy were found when the TMSC was stratified in groups. All groups demonstrated similar levels of pregnancy rates, even those with TMSC below 1 million/ml. Only when TMSC was divided into two groups it became apparent that TMSC ≥ 5 million/ml was significantly associated with the occurrence of pregnancy. Although these findings are important during counselling, they do not support any clear limits for TMSC levels before attempting TI.

Sample selection should be carefully conducted in order to exclude those couples who would be the least likely to get pregnant with TI, rather than those who would most benefit from it. Within a patient-friendly ART approach, cost-effectiveness, access to treatment, minimal risk and minimal burden should always be taken into account (Pennings & Ombelet, 2007). Only couples who had been trying to conceive for at least one year of regular intercourse without success should be considered for TI. According to the NICE guidelines (2013) couples facing unexplained and mild male factor infertility should be advised to keep trying to conceive for a total of 2 years before any IVF treatment should be considered (NICE, 2013). Previous studies have also reported on couples with unexplained subfertility being able to conceive spontaneously with no specific treatment (Snick *et al*., 2008; Farquhar *et al*., 2011).

Couples must undergo a thorough investigation of their medical history and sexual life routine, followed by complete physical examination. Provided the exams do not show any important contraindication, TI can then be considered as the first-line treatment. In the present study, SFH and AMH levels found demonstrated that participating women had a good ovarian reserve and were capable of getting pregnant. The data also showed that for every additional unit of AMH, the chances of pregnancy increased by 8.30%, clearly indicating the importance of this marker. Thus, the present findings are important to provide couples with more educated information concerning their chances of pregnancy with TI. Furthermore, it is always important to remember that apart from providing an opportunity to couples to conceive with minimal intervention, TI can also offer them time to get used to the ART interventions, especially concerning the response to ovulatory induction, and the medical team with useful information in the sequence of the treatment.

The findings of the present paper need to be interpreted based on the limitations of the study. The main limitation concerns the fact that this is study is retrospective in nature. Ideally, well-designed prospective studies should be conducted taking into consideration not only the clinical pregnancy results, but also live birth rates. Unfortunately, live birth rate was unavailable as couples treated in our service are referred to their regular doctors for their prenatal follow-up.

## CONCLUSION

Taking into consideration the results and limitations of the present study, it is possible to conclude that TI can be considered as the first-line treatment option for selected couples with unexplained subfertility before more traumatic and costly IVF treatments are considered. The findings within this study can assist doctors to conduct a more educated counselling concerning the chances patients have to get pregnant with TI.
